# Development of a heart rate variability and complexity model in predicting the need for life-saving interventions amongst trauma patients

**DOI:** 10.1186/s41038-019-0147-2

**Published:** 2019-04-18

**Authors:** Aravin Kumar, Nan Liu, Zhi Xiong Koh, Jayne Jie Yi Chiang, Yuda Soh, Ting Hway Wong, Andrew Fu Wah Ho, Takashi Tagami, Stephanie Fook-Chong, Marcus Eng Hock Ong

**Affiliations:** 1 Yong Loo Lin School of Medicine, National University of Singapore, Singapore, Singapore; 2 Health Services Research Centre, Singapore Health Services, Academia, 20 College Road, Singapore, 169856 Singapore; 3 Duke-NUS Medical School, National University of Singapore, Singapore, Singapore; 4 Department of Emergency Medicine, Singapore General Hospital, Singapore, Singapore; 5 Department of General Surgery, Singapore General Hospital, Singapore, Singapore; 6 Department of Emergency and Critical Care Medicine, Nippon Medical School, Tokyo, Japan; 7 Health Services Research Unit, Singapore General Hospital, Singapore, Singapore

**Keywords:** Triage trauma score, Heart rate variability, Heart rate complexity, Life-saving interventions

## Abstract

**Background:**

Triage trauma scores are utilised to determine patient disposition, interventions and prognostication in the care of trauma patients. Heart rate variability (HRV) and heart rate complexity (HRC) reflect the autonomic nervous system and are derived from electrocardiogram (ECG) analysis. In this study, we aimed to develop a model incorporating HRV and HRC, to predict the need for life-saving interventions (LSI) in trauma patients, within 24 h of emergency department presentation.

**Methods:**

We included adult trauma patients (≥ 18 years of age) presenting at the emergency department of Singapore General Hospital between October 2014 and October 2015. We excluded patients who had non-sinus rhythms and larger proportions of artefacts and/or ectopics in ECG analysis. We obtained patient demographics, laboratory results, vital signs and outcomes from electronic health records. We conducted univariate and multivariate analyses for predictive model building.

**Results:**

Two hundred and twenty-five patients met inclusion criteria, in which 49 patients required LSIs. The LSI group had a higher proportion of deaths (10, 20.41% vs 1, 0.57%, *p* < 0.001). In the LSI group, the mean of detrended fluctuation analysis (DFA)-α1 (1.24 vs 1.12, *p* = 0.045) and the median of DFA-α2 (1.09 vs 1.00, *p* = 0.027) were significantly higher. Multivariate stepwise logistic regression analysis determined that a lower Glasgow Coma Scale, a higher DFA-α1 and higher DFA-α2 were independent predictors of requiring LSIs. The area under the curve (AUC) for our model (0.75, 95% confidence interval, 0.66–0.83) was higher than other scoring systems and selected vital signs.

**Conclusions:**

An HRV/HRC model outperforms other triage trauma scores and selected vital signs in predicting the need for LSIs but needs to be validated in larger patient populations.

**Electronic supplementary material:**

The online version of this article (10.1186/s41038-019-0147-2) contains supplementary material, which is available to authorized users.

## Background

Trauma is well reported to be one of the leading causes of death amongst patients under the age of 45 [1, 2]. Trauma care and subsequent outcomes are time-sensitive, and providing early definitive care significantly decreases mortality [3, 4], making these patients an important demographic of focus for emergency physicians and first line healthcare professionals. Proper triage can be the difference between life and death in these scenarios, in both pre-hospital and emergency department care. Pre-hospital triage has proven to be important, with adequate trauma centre referrals lowering the overall risk of death by up to 25% [5, 6]. Emergency department triage also ensures critical life-saving interventions can be provided to those who need it the most. To aid personnel in these time-scarce moments, trauma scores have been developed for both pre-hospital triage [5] and emergency clinical decision-making [4].

Several trauma scores have been developed over the years. The first adopted scores were the Trauma Score (TS) [7], which is based on physiological parameters, and the Injury Severity Score (ISS) [8], which is based on the degree of anatomical injuries as described by the Abbreviated Injury Scale (AIS) [9]. There was then increased recognition that the physiological derangements in trauma patients were capable of drastically influencing their outcomes, leading to the amalgamation of physiological and anatomical parameters, such as in the Trauma Injury Severity Score (TRISS) [7, 10, 11]. The addition of physiological components gained widespread popularity as they provided further insight into the ability to predict outcomes such as mortality after trauma [12–15]. Stand-alone physiological scores emerged for easy utilisation in the field and emergency room setting, compared to the complex and time-consuming nature of anatomical scores [16].

Over the past few decades, new scoring systems have emerged such as the Mechanism, Glasgow Coma Scale (GCS), Age, Arterial Pressure (M-GAP) score [5, 17], GCS, Age and Systolic Blood Pressure (GAP) score [4], and the Modified Early Warning Score (MEWS) [18]. Their similarities lie in the ease of computing these scores in terms of easily available vital parameters and clinical scores, and hence have a clear advantage over anatomical trauma scores. Amongst physiological trauma scores, the Triage-Revised Trauma Score (T-RTS) [3] is the most popular.

Heart rate variability (HRV) and heart rate complexity (HRC) are electrocardiogram (ECG) derivatives that act as a measure of autonomic dysfunction, which have been explored in recent studies. HRV and HRC are based on RR interval variations [19], which refer to the variation between heartbeats, with lower HRV/HRC parameters being linked to increased mortality and morbidity in trauma [19–22]. Gauging the extent of autonomic dysfunction can have a direct impact on the treatment and care in these trauma patients, who may otherwise have normal or only slightly deranged vital signs, due to physiological compensatory mechanisms. When these compensatory mechanisms fail, and conventional vital signs crash, it is usually due to cardiovascular decompensation, and may be too late to intervene [23]. This unique quality of HRV/HRC analyses has led to interest in its application in pre-hospital triage and emergency clinical decision making; interest which has been bolstered by a recent study showing that a model combining HRV/HRC parameters and traditional vital signs outperformed vital signs alone at predicting the need for life-saving interventions (LSIs) and mortality [24]. HRV/HRC analyses give us insight into the physiological function of patients; physiological function is then directly correlated to the severity of injury and correspondingly, the need for LSIs.

In this study, we aim to close the gaps in knowledge regarding the relevance of real-time HRV/HRC analysis in the emergency department setting for critical decision making, by studying if previously reported real-time HRV/HRC analyses results are reproducible in our population. HRV/HRC analysis has not seen wide implementation yet, and our study aims to explore reasons for and against such implementation. We aimed to develop a model incorporating HRV and HRC to predicting the need for LSIs within 24 h of emergency department presentation and compare it with several triage trauma scores (T-RTS, MGAP, GAP, MEWS) and selected vital signs with a hypothesis that our unique model will outperform other pre-existing triage trauma scores.

## Methods

### Patients recruitment

The Singapore General Hospital (SGH) is a tertiary care, public hospital in Singapore, classified as a Level 1 trauma centre, with round-the-clock emergency medicine specialist coverage and trauma activation code team. Emergency medical services are provided by the Singapore Civil Defence Force (SCDF) fleet of ambulances, activated by a central dispatching system. SCDF conveys patients to the public hospital nearest to the incident location, based on defined geographical locations, in what is known as a catchment zone policy [25]. Transport to the nearest hospital is largely independent of trauma severity and triage, in view of the widespread capability of emergency departments island-wide in dealing with trauma [26]. Adult trauma patients (≥ 18 years of age) presenting at the emergency department of SGH between October 2014 and October 2015 were included in this study. Patients who had non-sinus rhythm (asystole, ventricular or supraventricular arrhythmias) were excluded as these can lead to inaccuracies in HRV/HRC computation. ECGs that contained artefacts and/or ectopics in more than 30% of the entire ECG reading were also excluded. This study obtained ethics approval from SingHealth Institutional Review Board.

### Data collection

We collected patient demographic information, vital signs, laboratory results and outcome information retrospectively from electronic health records. These parameters were then used to calculate selected triage trauma scores. The triage trauma scores that we included in this study are T-RTS, MEWS, M-GAP and GAP. The calculation of the scores was based on presenting vital signs and initial laboratory results for standardisation. Lower scores signified greater physiological dysfunction and severity in T-RTS, M-GAP and GAP. For MEWS, the higher the score is, the greater the physiological dysfunction. Details of the various scoring criteria for the trauma scores are listed in Additional file 1: Table S1-Table S4.

Routine ECG evaluations are performed on arrival for all trauma patients presenting in the emergency department, in accordance with trauma pathways. These routine ECG readings of the patients allocated for HRV analysis were retrieved from the ZOLL X Series Monitor defibrillator (ZOLL Medical Corporation, MA, USA). The ECG readings of 5 min segments were processed and analysed using the Kubios HRV Software (KUBIOS HRV, University of Eastern Finland, Kuopio, Finland) [27]. Beat annotations were obtained by automated analysis by the software, and manual review. In this study, we conducted time domain, frequency domain and non-linear analyses. Time domain analysis and frequency domain analysis are regarded as HRV parameters, and non-linear analysis is regarded as HRC parameters. The HRV and HRC parameters were analysed as continuous variables.

### Outcomes

Our primary outcome was the need for LSIs, within 24 h of emergency department presentation, and this is defined as any intervention that would directly prolong the life of the patient. These include blood transfusions, endotracheal intubations, surgical interventions for trauma, cardioversion, chest tube placement, cardiopulmonary resuscitation (CPR), cricothyroidotomy, thoracotomy, angiography with or without embolisation, needle decompression, tourniquet application, use of vasoactive medications and hyperosmolar fluid therapy [22]. Patients who received more than one LSI were not double-counted in our study.

### Statistical analysis

Statistical analysis was performed using SPSS version 20.0 (SPSS, IBM Corporation, IL, USA). All continuous variables were checked for normality by considering the Shapiro-Wilk test [19]. For parameters with normal distribution, means and standard deviations were computed, and univariate analysis was conducted with Student’s *t* test. Parameters that were not normally distributed are presented as medians and interquartile ranges, and univariate analysis was conducted with Mann-Whitney *U* test. Pearson’s chi-squared test was performed for all categorical variables. Statistically significant was considered as *p* < 0.05.

Univariate analysis was performed to compare HRV parameters between the LSI and non-LSI groups, reflecting demographics, vital signs, laboratory results and outcome parameters. Multivariate regression analysis with backward Wald selection was performed for HRV parameters with a univariate *p* value < 0.2. Patient demographics and vitals were added as covariates in the multivariate regression analysis, with clinical relevance in mind. Assumptions for the regression analysis were tested and met. Specifically sample size was found to be adequate for the regression analysis as per Green [28] (recommended minimum sample size is 186), no significant causal association was determined and there was no collinearity as variance inflation factor (VIF) scores were well below 10, and tolerance scores above 0.2 (statistics are 4.90 and 0.63 respectively). Using the results of the multivariate regression analysis, we built a model incorporating vital signs and HRV/HRC parameters. We then generated the receiver operating characteristic (ROC) curves for this model, selected vital signs as well as for other triage trauma scores. We calculated the areas under the ROC curve (AUC), sensitivities, specificities, positive and negative predictive values.

## Results

**Fig. 1:**
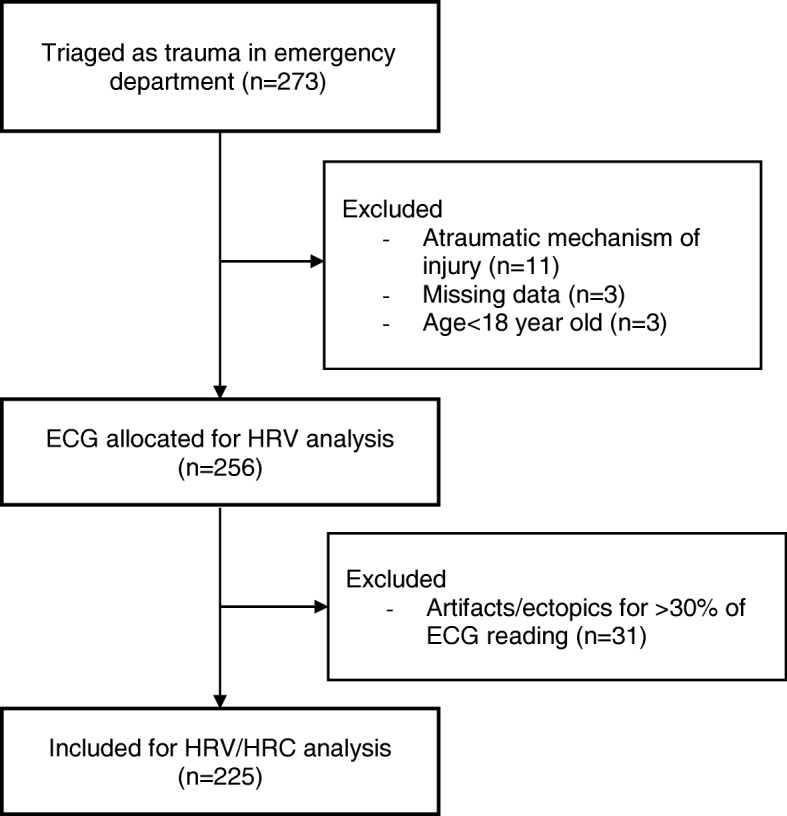
Patient breakdown for heart rate variability (HRV) and heart rate complexity (HRC) analysis. *ECG* electrocardiogram

Two hundred and seventy-three patients were triaged as trauma patients in the emergency department. Forty-eight patients were excluded (11 non-trauma patients, 3 underage patients, 3 patients with missing data and 31 patients with artefacts and/or ectopics in more than 30% of their ECG readings) (Fig. 1). There were no patients who presented with ventricular and supraventricular arrhythmias, and data was not collected for patients who presented to the emergency department with asystole. Two hundred and twenty-five patients were included for HRV/HRC analysis.

### Demographics

Patient demographics and incidences of co-morbidities are shown in Table 1, of whom 49 patients (21.77%) required LSIs. The most common LSI was the need for surgical management 24 h after trauma (35, 71.42%), followed by blood and blood product transfusions (16, 32.65%) and endotracheal intubation (10, 20.40%). Other LSIs that were administered in this study population were cardioversion (1, 0.02%), chest tube placement (3, 0.06%), CPR (1, 0.02%) and hyperosmolar fluid therapy (1, 0.02%). Comparing patients who received LSIs with those who did not, the LSI patients were younger on average, but this was not statistically significant. We also found that there were a significantly higher proportion of males (42, 85.71% vs 115, 65.34%; *p* = 0.006) in the patients requiring LSIs. There were no other significant differences in terms of ethnicity and co-morbidities.

**Table 1:** Characteristics of patient demographics, co-morbidities, mechanisms of traumatic injury, outcomes, life-saving interventions (LSI), vital signs, laboratory results and triage trauma scores

	LSI (*n* = 49)	Non-LSI (*n* = 176)	*P* value
Patient demographics			
Age (median, IQR)	39, 27–53	44, 31–62	0.051
Age> 56 (*n*, %)	9, 18.36%	62, 35.23%	0.025
Male (*n*, %)	42, 85.71%	115, 65.34%	0.006
Chinese (*n*, %)	28, 57.14%	105, 59.66%	0.751
Ischemic heart disease (*n*, %)	0, 0.00%	9, 5.11%	0.106
Diabetes mellitus (*n*, %)	3, 0.06%	21, 11.93%	0.244
Hypertension (*n*, %)	7, 14.29%	36, 20.45%	0.331
Hyperlipidemia (*n*, %)	3, 0.06%	26, 14.77%	0.110
Congestive cardiac failure (*n*, %)	1, 0.02%	3, 0.02%	0.875
Cancer (*n*, %)	0, 0.00%	13, 0.07%	0.052
Respiratory disease (*n*, %)	2, 0.04%	10, 0.06%	0.659
Renal disease (*n*, %)	0, 0.00%	6, 0.03%	0.190
Mechanisms of traumatic injury			
Fall (*n*, %)	7, 14.29%	87, 49.43%	< 0.001
Road traffic accident (*n*, %)	19, 38.78%	46, 26.14%	0.084
Burns (*n*, %)	9, 18.37%	15, 0.09%	0.048
Others (*n*, %)	14, 28.57%	28, 15.91%	0.044
Outcomes			
Intensive care unit admission (*n*, %)	22, 44.90%	5, 2.84%	< 0.001
Death (*n*, %)	10, 20.41%	1, 0.57%	< 0.001
Overall stay (median, IQR)	21, 9–106	3, 2–15	< 0.001
LSI (*n*, %)	49, 21.78%		
Operating theatre within 24 h, for trauma (*n*, %)	35, 71.43%		
Endotracheal intubation (*n*, %)	10, 20.41%		
Blood transfusion (*n*, %)	16, 32.65%		
Vital signs			
Heart rate, beats per minute (mean ± SD)	93.49 ± 21.28	85.42 ± 16.32	0.020
Respiratory rate, breaths per minute (median, IQR)	18.00, 14–19	18.00, 18–18	0.396
Temperature, degrees Celsius (median, IQR)	36.50, 31.3–36.7	36.70, 36.1–36.9	0.972
Systolic blood pressure, mmHg (mean ± SD)	126.14 ± 29.59	138.45 ± 24.76	0.238
Diastolic blood pressure, mmHg (mean ± SD)	75.41 ± 19.31	77.40 ± 14.84	0.106
Oxygen saturation, % (median, IQR)	99, 98–100	100, 98–100	0.397
Glasgow coma scale (median, IQR)	15, 3–15	15, 15–15	< 0.001
Pain score, out of 10 (median, IQR)	6, 0–9	7, 0–10	0.798
Laboratory results			
Haemoglobin (mean ± SD)	13.97 ± 2.55 (*n* = 49)	13.48 ± 2.38 (*n* = 151)	0.852
pH (median, IQR)	7.00, 7.00–7.31 (*n* = 17)	7.36, 7.25–7.41 (*n* = 22)	0.038
Lactate (median, IQR)	2.7, 2.1–3.6 (*n* = 23)	2.2, 1.8–3.3 (*n* = 38)	0.015
Triage trauma scores			
T-RTS (median, IQR)	12, 8–12	12, 12–12	< 0.001
MEWS (median, IQR)	1, 1–3	1, 1–2	< 0.001
M-GAP (median, IQR)	25, 17–26	27, 22–29	0.002
GAP (median, IQR)	22, 12–24	24, 19–24	0.011

*IQR* interquartile range, *SD* standard deviation, *T*-*RTS* Triage Revised Trauma Score, *MEWS* Modified Early Warning System, *M*-*GAP* Mechanism of injury, Glasgow Coma Scale, Age and Arterial blood pressure, *GAP* Glasgow Coma Scale, Age and Systolic blood pressure

We analysed the mechanisms of injury in our patients (Table 1) and found that the most common mechanism was a fall (94 patients, 41.77%). Sixty-five patients (28.88%) were involved in a road traffic accident (RTA) and 24 patients (10.67%) had injuries related to burns. Forty-two patients (18.66%) presented with other mechanisms of injury, including work-related injuries (2 patients), assault (1 patient), trauma from falling objects (1 patient) and maritime accidents (2 patients). Comparing the two groups, we found that there was a significantly lower number of patients involved in falls in the LSI group as compared to the non-LSI group (7, 14.28% vs 87, 49.43%; *p* < 0.001). There was a greater proportion of LSI patients involved in RTA, but this was not significant (19, 38.77% vs 46, 26.13%; *p* = 0.084). A significantly higher proportion of burns patients required LSIs (9, 18.36% vs 15, 0.09%; *p* = 0.048). In total, 27 patients (12.00%) required intensive care unit admission, 11 patients perished (4.9%) and the median length of hospitalisation was 5 days. In terms of these outcomes, patients in the LSI group fared worse, with a greater incidence of intensive care unit admission (22, 44.90% vs 5, 2.84%; *p* < 0.001), death (10, 20.41% vs 1, 0.57%; *p* < 0.001) and a longer hospitalisation duration (21 days, interquartile range [IQR] 9–106 vs 3 days, IQR 2–15; *p* < 0.001).

### Univariate analysis

Univariate analysis was carried out for vital signs and laboratory results (Table 1), and HRV/HRC parameters (Table 2). Patients in the LSI group had significantly higher heart rate readings (93.49 beats per minute ± 21.28 vs 85.42 beats per minute ± 16.32, *p* = 0.02). There was also a significant difference in the GCS scores (15, IQR 3–15 vs 15, IQR 15–15; *p* < 0.001), with LSI patients having a greater variability of GCS scores, with more scores which were less than 15, as compared to the non-LSI group. There were no significant differences in terms of the systolic (126.14 mmHg ± 29.59 vs 138.45 mmHg ± 24.76, *p* = 0.24) and diastolic (75.41 mmHg ± 19.31 vs 77.40 mmHg ± 14.84; *p* = 0.106) blood pressures. Blood pH levels (7.00, IQR 7.00–7.31 vs 7.36, IQR 7.25–7.41; *p* = 0.038) and lactate levels (2.7, IQR 2.1–3.6 vs 2.2, IQR 1.8–3.3; *p* = 0.015) were more deranged amongst LSI patients as compared to non-LSI patients. There was no significant difference in terms of serum haemoglobin levels between the two groups.

**Table 2:** Univariate analysis of heart rate variability and complexity indices in associations with the need for life-saving interventions (LSI) amongst trauma patients

	LSI (*n* = 49)	Non-LSI (*n* = 176)	*P* value
Heart rate variability time domain analysis			
aRR (mean ± SD)	704.58 ± 166.48	746.76 ± 141.49	0.302
sdRR (mean ± SD)	30.86 ± 31.20	34.71 ± 28.55	0.982
avHR (median, IQR)	97.33, 85.59–122.96	88.96, 83.47–96.40	0.029
sdHR (median, IQR)	2.03, 1.71–3.32	3.08, 1.88–4.53	0.899
RMSSD (mean ± SD)	17.03 ± 26.46	35.12 ± 148.41	0.278
NN50 (median, IQR)	0.00, 0.00–2.00	0.00, 0.00–13.75	0.043
pNN50 (median, IQR)	0.00, 0.00–0.45	0.00, 0.00–3.85	0.051
TINN (mean ± SD)	122.56 ± 93.47	150.75 ± 137.51	0.399
Heart rate variability frequency domain analysis			
VLF (median, IQR)	58.50, 41.75–218.25	243.00, 132.00–396.00	0.416
LF ms^2^ (median, IQR)	15.50, 3.00–217.00	100.00, 60.00–262.00	0.072
HF ms^2^ (median, IQR)	4.50, 0.75–25.00	44.00, 21.00–167.00	0.011
LF norm (median, IQR)	89.05, 67.98–92.90	78.00, 50.30–84.00	0.128
HF norm (median, IQR)	10.95, 7.03–31.98	21.00, 16.00–49.30	0.128
LF/HF (median, IQR)	8.11, 3.32–13.26	4.52, 1.02–5.62	0.147
TP (median, IQR)	76.00, 46.50–504.25	454.00, 275.00–713.00	0.175
Heart rate complexity non-linear analysis			
Poincare plot SD1(mean ± SD)	12.01 ± 18.76	17.49 ± 25.03	0.257
Poincare plot SD2 (mean ± SD)	41.34 ± 40.65	43.42 ± 31.65	0.545
Approximate entropy (mean ± SD)	1.00 ± 0.25	1.09 ± 0.15	0.001
Sample entropy (mean ± SD)	1.13 ± 0.45	1.39 ± 0.38	0.144
DFA-α1 (mean ± SD)	1.24 ± 0.39	1.12 ± 0.31	0.045
DFA-α2 (median, IQR)	1.09, 0.93–1.68	1.00, 0.89–1.11	0.027

*IQR* interquartile range, *SD* standard deviation, *aRR* average width of the RR intervals in electrocardiograms, *sdRR* standard deviation of all RR intervals in electrocardiograms, *avHR* mean of the instantaneous heart rate in electrocardiograms, *sdHR* standard deviation of the instantaneous heart rate in electrocardiograms, *RMSSD* root mean square of differences between adjacent RR intervals in electrocardiograms, *NN50* number of consecutive RR intervals differing by more than 50 ms in electrocardiograms, *pNN50* percentage of consecutive RR intervals differing by more than 50 ms in electrocardiograms, *TINN* baseline width of a triangle fit into the RR interval histogram using a least squares technique in electrocardiograms, *VLF* very low frequency power in electrocardiograms, *LF* low frequency power in electrocardiograms, *HF* high frequency power in electrocardiograms, *norm* normalised, *LF*/*HF* ratio of LF power to HF power in electrocardiograms, *TP* total power derived from variance of all RR intervals in electrocardiograms, *DFA* detrended fluctuation analysis

As shown in Table 2, amongst the HRV/HRC parameters, we found that mean of the instantaneous heart rate in electrocardiograms (avHR) (97.33, IQR 85.59–122.96 vs 88.96, IQR 83.47–96.40; *p* = 0.029) which measures average heart rate on ECG readings was significantly elevated in the LSI group as compared to the non-LSI group. In the time domain analysis segment, we also noticed that number of consecutive RR intervals differing by more than 50 ms in electrocardiograms (NN50), a measure of variability of RR intervals in ECG readings, was significantly different, with the non-LSI group showing greater variability. In the frequency domain analysis, we found that high frequency (HF) that measures power in the high frequency range was significantly decreased in the LSI group. In the non-linear analysis, we found that approximate entropy (ApEn) (1.00 ± 0.25 vs 1.09 ± 0.15, *p* = 0.001), which measures approximate entropy, was significantly lower in the LSI group. Detrended fluctuation analysis (DFA)-α1 and DFA-α2 were significantly higher in the LSI group as compared to the non-LSI group, and these are readings of DFA.

We compared triage trauma scores between the LSI and non-LSI group, in Table 1. Amongst the 225 patients, the median T-RTS score was 12 (IQR 12–12), the median MEWS score was 1 (IQR 1–1), the median M-GAP score was 27 (IQR 24–29) and the median GAP score was 23 (IQR 21–24). As expected, there were significant differences between the LSI group and non-LSI group in all the scores, with the LSI group having a greater proportion of deranged scores.

### Multivariate analysis

**Fig. 2:**
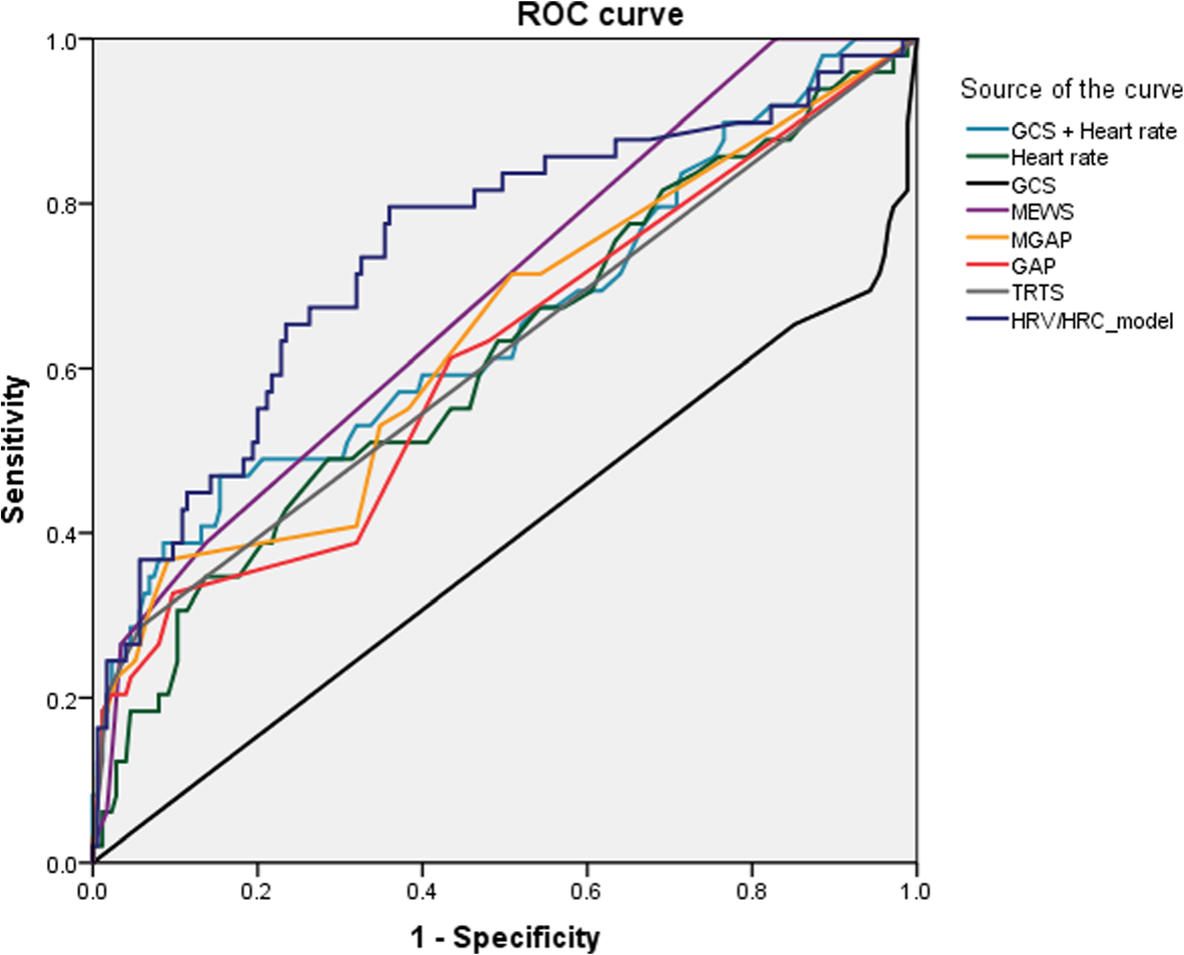
Receiver operating characteristic (ROC) curves for heart rate variability/heart rate complexity (HRV/HRC) model, selected vital signs and triage trauma scores. *GCS* Glasgow Coma Scale, *MEWS* modified early warning system, *MGAP* Mechanism of injury, Glasgow Coma Scale, Age and Arterial blood pressure, *GAP* Glasgow Coma Scale, Age and Systolic blood pressure, *T*-*RTS* Triage Revised Trauma Score

Multivariate logistic regression analysis (Table 3) included patient demographics, vital signs and HRV/HRC indices, which then revealed that GCS (odds ratio (OR) 0.756, 95% confidence interval (CI) 0.660–0.864; *p* < 0.001), DFA-α1 (OR 3.932, 95% CI 1.256–12.314; *p* = 0.019) and DFA-α2 (OR 5.200, 95% CI 1.060–25.504; *p* = 0.042) were significant. As shown in Fig. 2, the ROC curve for our HRV/HRC model was compared with the ROC curves of the other triage trauma scores as well as GCS, heart rate and a combination of GCS and heart rate. Table 4 presents the corresponding predictive performance of various models and scores. The HRV/HRC model had a greater AUC value (0.75, 95% CI: 0.66–0.83) as compared to the other triage trauma scores and that of GCS, heart rate and a combination of GCS and heart rate. The HRV/HRC model also had a higher sensitivity as compared to the other triage trauma scores and selected vital signs. However, the HRV/HRC model had a lower specificity compared to MEWS and GAP. It had a positive predictive value that was larger than M-GAP and GAP, but lower than MEWS. The HRV/HRC model achieved the greatest negative predictive value.

**Table 3:** Multivariate logistic regression analysis for predicting the need for life-saving interventions amongst trauma patients

	OR (95% CI)	*P* value
All variable		
Age	1.026 (0.987–1.066)	0.200
Age greater than 56	0.363 (0.079–1.674)	0.194
Systolic blood pressure	0.989 (0.972–1.006)	0.201
Heart rate	1.021 (0.997–1.044)	0.085
Glasgow Coma Scale	0.819 (0.699–0.960)	0.014
avHR	1.002 (0.973–1.031)	0.916
NN50	0.996 (0.958–1.036)	0.840
pNN50	1.043 (0.903–1.204)	0.570
LF	1.000 (0.999–1.002)	0.328
HF	0.999 (0.999–1.000)	0.224
LF norm	0.817 (0.574–1.162)	0.261
HF norm	0.842 (0.590–1.199)	0.340
LF/HF	1.000 (0.998–1.002)	0.837
Approximate entropy	1.866 (0.060–58.263)	0.723
Sample entropy	0.364 (0.056–2.366)	0.290
DFA1	10.157 (1.621–63.659)	0.013
DFA2	3.758 (0.653–21.629)	0.138
Variables after backward Wald selection		
Glasgow Coma Scale	0.756 (0.660–0.864)	< 0.001
DFA-α1	3.932 (1.256–12.314)	0.019
DFA-α2	5.200 (1.060–25.504)	0.042

*OR* odds ratio, *CI* confidence interval, *avHR* mean of the instantaneous heart rate in electrocardiograms, *NN50* number of consecutive RR intervals differing by more than 50 ms in electrocardiograms, *pNN50* percentage of consecutive RR intervals differing by more than 50 ms in electrocardiograms, *LF* low frequency power in electrocardiograms, *HF* high frequency power in electrocardiograms, *norm* normalised, *LF*/*HF* ratio of LF power to HF power in electrocardiograms, *TP* total power derived from variance of all RR intervals in electrocardiograms, *DFA* detrended fluctuation analysis

**Table 4:** Comparison of predictive performance in receiver operating characteristic analysis

	HRV/HRC model	T-RTS	MEWS	M-GAP	GAP	GCS	Heart rate	GCS + heart rate
Cut-off	0.18	12	2	27	22	15	84.5	0.18
AUC (95% CI)	0.75 (0.66–0.83)	0.62 (0.53–0.71)	0.69 (0.60–0.78)	0.64 (0.55–0.73)	0.61 (0.52–0.70)	0.39 (0.29–0.48)	0.61 (0.52–0.71)	0.65 (0.56–0.75)
Sensitivity %, (95% CI)	79.6% (68.3–90.9)	28.6% (15.9–41.2)	26.5% (14.2–38.9)	55.1% (41.2–69.0)	38.8% (25.1–52.4)	65.3% (58.9–78.2)	63.3% (48.3–76.6)	57.1% (42.2–71.2)
Specificity %, (95% CI)	63.1% (55.9–70.2)	94.3% (90.9–97.7)	96.6% (93.9–99.3)	61.4% (54.2–68.6)	68.2% (61.3–75.1)	14.9% (11.3–21.9)	51.0% (42.9–58.2)	61.0% (52.6–67.5)
PPV %, (95% CI)	37.5% (28.2–46.8)	58.3% (38.6–78.1)	68.4% (47.5–89.3)	28.4% (19.4–37.5)	25.3% (15.5–35.2)	38.6% (27.3–51.4)	26.3% (21.5–31.6)	28.6% (22.8–35.1)
NPV %, (95% CI)	91.7% (86.8–96.6)	82.6% (77.3–87.8)	82.5% (77.3–87.7)	83.1% (76.6–89.5)	80.0% (73.6–86.4)	82.3% (79.0–85.2)	83.2% (76.9–88.0)	83.5% (78.1–87.7)

*HRV* heart rate variability, *HRC* heart rate complexity, *T*-*RTS* Triage Revised Trauma Score, *MEWS* Modified Early Warning System, *M*-*GAP* Mechanism of injury, Glasgow Coma Scale, Age and Arterial blood pressure, *GAP* Glasgow coma scale, Age and Systolic blood pressure, *AUC* area under the curve, *CI* confidence interval, *PPV* positive predictive value, *NPV* negative predictive value, *GCS* Glasgow Coma Scale

## Discussion

In this study, we constructed a model incorporating vital signs and HRV/HRC parameters for the prediction of the need for LSIs within 24 h of trauma patients presenting to the emergency department. In comparison, the HRV/HRC model outperformed several conventional triage trauma scores as well as common vital signs in terms of AUC value, sensitivity, positive predictive value and negative predictive value.

Common vital signs and 12-lead ECG variables are routinely monitored for clinical decision making and risk stratification in emergency departments [18], and HRV and HRC parameters are increasingly being incorporated for clinical decision making as well [29, 30]. The simplicity of our model which incorporates such routine vital signs and HRV/HRC parameters allows for its real-time use and application in the field and emergency room.

We found that the systolic blood pressures do not significantly differ in the LSI group and the non-LSI group. This is likely due to the natural physiological compensatory mechanisms that work to maintain normal systolic blood pressure as previously theorised. This lack of obvious difference in blood pressure underlines the importance of HRV/HRC analysis, which provides a window into the level of autonomic dysfunction which is directly related to patient outcomes.

We also showed that in LSI patients, there were comparatively lower HRV parameters (NN50 and HF) and HRC parameters (ApEn). There were higher HRC parameters of DFA-α1 and DFA-α2, compared to the non-LSI group as well. These parameters describe decreased parasympathetic stimulation [30–34], as well as a greater degree of autonomic dysfunction [35–37] in the LSI group. Autonomic dysfunction, measured by low sample entropy and ApEn parameters, are well documented to be associated with cardiovascular dysfunction, severity of disease, need for LSI and mortality in trauma patients [19, 22, 30, 35, 38, 39]. In our study, we similarly found that the LSI group patients had lower mean ApEn and sample entropy  parameters as compared to the non-LSI group, but this was only statistically significant for the ApEn metric.

Detrended fluctuation analysis is a method that allows for detection of short- and long-range correlations within the data over time [40]. This is based on the principle that healthy heart rate fluctuations are fractal in nature, which is to say that there are ‘self-similar’ fluctuations over periods of time ranging from seconds to hours. This complex type of variability means that there are long-range power-law correlations and indicates that the fluctuations in heart rate are not influenced just by the most recent value but also by more remote events (a sort of ‘memory event’) [41]. Lower DFA values have then been hypothesised to suggest a breakdown in these fractal scaling properties and lower DFA values have been seen in previous studies in patients requiring LSIs, patients that did not survive and in patients who developed myocardial infarctions and developed atrial fibrillation [22, 30, 39, 41]. In our study though, we found that patients who required LSIs had higher DFA parameters, and this was found to be statistically significant. This high DFA values were also found to be independent predictors of LSI on multivariate analysis.

To interpret these results then requires an understanding of the significance of DFA. DFA essentially measures the self-similarity of fractal processes by quantification of the short- and long-term correlations in the data [40]. Normal RR interval signals will feature a certain amount of such correlations, which render the overall dynamics of the system to be neither completely random nor completely organised, resulting in a normal DFA value of 1. Deviations from this value in either direction are then abnormal [36]. In this study, we have shown that DFA values tend to be greater than 1.00 in the LSI group as compared to the non-LSI group, and this can be interpreted to mean that correlations still exist, but they are no longer a power law [42]. We are satisfied with this aberration from the normal value to be representative of significant abnormalities in DFA which can then predict the need for LSI, though we note that there are no significant studies that show such increased DFA values.

Multivariate analysis showed that GCS, DFA-α1 (short-term exponent) and DFA-α2 (intermediate to long-term exponent) are independent predictors of LSI. The inclusion of both DFA-α1 and DFA-α2 into a model with GCS outperforms other triage trauma scores and underlines the usefulness of these ECG-derived metrics.

Our study highlights the usefulness of HRV/HRC analyses in the emergency setting and we believe that it helps to pave the way for use of this model in a pre-hospital setting or in settings that facilitate remote monitoring and triage. This comes on the back of studies that have questioned the use of HRV and HRV/HRC metrics for emergency and pre-hospital care, in the identification of haemorrhaging patients [20], and due to interindividual variability [23, 43] respectively. Studies have also shown that standard clean vital signs may be better than HRV and HRC metrics at predicting LSI [20, 44], with greater AUROC values than HRV models. However, we showed that our HRV/HRC model outperformed models of GCS, heart rate and a combined model of GCS and heart rate in our study population. Our HRV/HRC model has outperformed both conventional vital signs and triage trauma scores, but there is a need for further studies and trials in HRV/HRC to demonstrate its efficacy and usefulness.

### Limitations

Our study was limited by the patient flow in a single centre, leaving room for future multicentre studies and trials to validate our model in predicting the need for LSI in larger, more diverse patient populations. Our study was also limited to patients presenting in the emergency department, with our model only having been validated on this group of patients. We are assuming that our model would produce similar results in other settings such as the pre-hospital setting or in the remote triage setting. The inter-individual variability in HRV/HRC parameters is another limitation [29]. Patients of different ages, races, genders, levels of fitness, amount of alcohol intake and many more may have different HRV parameter values, even if they are healthy individuals [45]. This inter-individual variability is then complicated by the ever-changing nature of the emergency department, in which conditions cannot be controlled. This sensitivity for individual differences may limit the reproducibility and applicability of our model in different patient populations in other emergency rooms around the world. Our study was also limited by 31 samples having to be excluded from further analyses on account of artefacts or ectopics on ECG readings. HRV/HRC parameters are derived from ECG readings, which are electrical signals picked up from cutaneous electrical leads on the patient’s body. In the unpredictable setting of trauma, and an ever-changing emergency room, environmental forces are unavoidable. These forces induce electromechanical artefacts and ectopic beats which lead to a substantial reduction in eligible ECG waveforms, that may preclude patients from HRV/HRC analysis [46]. This will likely remain to be a limitation in any study involving the care of patients in the pre-hospital and emergency care setting due to the unpredictability of setting and time critical nature of care for unstable patients [47]. Lastly, we also excluded the comparison with retrospective trauma scores in our study. Whilst physiological trauma scores are on the rise and are being used in the clinical setting more, retrospective trauma scores such as ISS and TRISS are still considered to be the gold standard of trauma severity scoring [48, 49]. There is then a need for future studies to compare our model with these retrospective trauma scores, in order to effectively comment on the benefits of HRV/HRC-based models in trauma.

## Conclusions

In this single-centre study of trauma patients presenting to the ED, our triage model that incorporates HRV/HRC parameters and conventional vital signs outperformed several triage trauma scores and conventional vital signs in predicting the need for LSI. By incorporating GCS and 12-lead ECG derivatives, our model is easily applicable and can be seamlessly incorporated into daily emergency department machines and processes. Our findings build upon a backbone of literature citing the relevance and applicability of HRV and HRC parameters in trauma patients. This technology can easily be integrated into currently existing defibrillator machines in other emergency rooms and unlocks future possibilities for use in the pre-hospital, remote triage and early triage settings. Furthermore, our study showcases the ability to incorporate HRV/HRC analysis into models with traditional vital signs to predict the need for LSI amongst trauma patients. However, whilst HRV/HRC analysis provides interesting insights into trauma care, there are also obstacles that this technology faces before widespread clinical implementation can be achieved.

## Additional file

Additional file 1: Triage trauma scores. **Table S1.** Triage revised-trauma score (T-RTS) [3]. **Table S2.** Mechanism of injury, Glasgow coma scale, age and arterial blood pressure (M-GAP) [5]. **Table S3.** Glasgow coma scale, age and systolic blood pressure (GAP) [4]. **Table S4.** Modified early warning score (MEWS) [18]. (DOCX 40 kb)

## Abbreviations

AIS: Abbreviated Injury Scale
AUC: Area under the curve
CPR: Cardiopulmonary resuscitation
DFA: Detrended fluctuation analysis
ECG: Electrocardiogram
GAP: Glasgow Coma Scale, Age and Systolic blood pressure
GCS: Glasgow Coma Scale
HF: High frequency
HRC: Heart rate complexity
HRV: Heart rate variability
ISS: Injury Severity Score
LF: Low frequency
LF/HF: Ratio of low frequency power to high frequency power
LSI: Life-saving interventions
MEWS: Modified Early Warning Score
MGAP: Mechanism, Glasgow Coma Scale, Age, Arterial Pressure
RMSSD: Root mean square of differences between adjacent RR intervals
ROC: Receiver-operating characteristic
RTA: Road traffic accident
SCDF: Singapore Civil Defence Force
SGH: Singapore General Hospital
TINN: Baseline width of a triangle fit into the RR interval histogram using a least squares
TP: Total power
TRISS: Trauma Injury Severity Score
T-RTS: Triage-Revised Trauma Score
TS: Trauma Score
VLF: Very low frequency
