# Supplementary material for: Development of a heart rate variability and complexity model in predicting the need for life-saving interventions amongst trauma patients
Source: Burns Trauma. 2019 Apr 18;7:12. doi: 10.1186/s41038-019-0147-2 (PMC6471773; doi:10.1186/s41038-019-0147-2)
Supplement: Supplementary file 1 — Triage trauma scores. Table S1. Triage revised-trauma score (T-RTS) [3]. Table S2. Mechanism of injury, Glasgow coma scale, age and arterial blood pressure (M-GAP) [5]. Table S3. Glasgow coma scale, age and systolic blood pressure (GAP) [4]. Table S4. Modified early warning score (MEWS) [18]. (DOCX 40 kb) [file 41038_2019_147_MOESM1_ESM.docx]

**Additional File 1**

**Triage trauma scores**

**Table S1 Triage Revised-Trauma Score (T-RTS) [**[**3**](#_ENREF_3)**]**

|  | 4 | 3 | 2 | 1 | 0 |
| --- | --- | --- | --- | --- | --- |
| Systolic Blood Pressure (mm Hg) | >89 | 76-89 | 50-75 | 1-49 | 0 |
| Respiratory Rate (Breaths per minute) | 10-29 | >29 | 6-9 | 1-5 | 0 |
| Glasgow Coma Scale | 15-13 | 12-9 | 8-6 | 5-4 | 3 |

**Table S2 Mechanism of Injury, Glasgow Coma Scale, Age and Arterial Blood Pressure (M-GAP) [**[**5**](#_ENREF_5)**]**

| **Variable** | **Score** |
| --- | --- |
| **Age** | |
| < 60 years of age | + 5 |
| > 60 years of age | 0 |
| **Glasgow Coma Scale (GCS)** | |
| GCS Value | 3-15 |
| **Mechanism of trauma** | |
| Blunt trauma | + 4 |
| Penetrating trauma | 0 |
| **Systolic Blood Pressure** | |
| > 120 mm Hg | + 5 |
| 60 – 120 mm Hg | + 3 |

**Table S3 Glasgow Coma Scale, Age and Systolic Blood Pressure (GAP) [**[**4**](#_ENREF_4)**]**

| **Variable** | **Score** |
| --- | --- |
| **Age** | |
| < 60 years of age | + 5 |
| > 60 years of age | 0 |
| **Glasgow Coma Scale (GCS)** | |
| GCS Value | 3-15 |
| **Systolic Blood Pressure** | |
| > 120 mm Hg | + 5 |
| 60 – 120 mm Hg | + 3 |

**Table S4 Modified Early Warning Score (MEWS) [**[**18**](#_ENREF_18)**]**

| **Variables** | **3** | **2** | **1** | **0** | **1** | **2** | **3** |
| --- | --- | --- | --- | --- | --- | --- | --- |
| Systolic Blood pressure (mm Hg) | <70 | 71–80 | 81–100 | 101–199 |  | ≥200 |  |
| Heart rate (bpm) |  | <40 | 41–50 | 51–100 | 101–110 | 111–129 | ≥130 |
| Respiratory rate (bpm) |  | <9 |  | 9–14 | 15–20 | 21–29 | ≥30 |
| Temperature (°C) |  | <35 |  | 35–38.4 |  | ≥38.5 |  |
| AVPU score |  |  |  | Alert | Reacting to voice | Reacting to pain | Unresponsive |
